# Health-Related Behaviors and Academic Achievement Among High School Students — United States, 2015

**DOI:** 10.15585/mmwr.mm6635a1

**Published:** 2017-09-08

**Authors:** Catherine N. Rasberry, Georgianne F. Tiu, Laura Kann, Tim McManus, Shannon L. Michael, Caitlin L. Merlo, Sarah M. Lee, Michele K. Bohm, Francis Annor, Kathleen A. Ethier

**Affiliations:** ^1^Division of Adolescent and School Health, National Center for HIV/AIDS, Viral Hepatitis, STD, and TB Prevention, CDC; ^2^These authors contributed equally to this report; ^3^Division of Population Health, National Center for Chronic Disease Prevention and Health Promotion, CDC; ^4^Division of Unintentional Injury Prevention, National Center for Injury Prevention and Control, CDC; ^5^Division of Violence Prevention, National Center for Injury Prevention and Control, CDC.

Studies have shown links between educational outcomes such as letter grades, test scores, or other measures of academic achievement, and health-related behaviors (*1*–*4*). However, as reported in a 2013 systematic review, many of these studies have used samples that are not nationally representative, and quite a few studies are now at least 2 decades old (*1*). To update the relevant data, CDC analyzed results from the 2015 national Youth Risk Behavior Survey (YRBS), a biennial, cross-sectional, school-based survey measuring health-related behaviors among U.S. students in grades 9–12. Analyses assessed relationships between academic achievement (i.e., self-reported letter grades in school) and 30 health-related behaviors (categorized as dietary behaviors, physical activity, sedentary behaviors, substance use, sexual risk behaviors, violence-related behaviors, and suicide-related behaviors) that contribute to leading causes of morbidity and mortality among adolescents in the United States (*5*). Logistic regression models controlling for sex, race/ethnicity, and grade in school found that students who earned mostly A's, mostly B’s, or mostly C’s had statistically significantly higher prevalence estimates for most protective health-related behaviors and significantly lower prevalence estimates for most health-related risk behaviors than did students with mostly D’s/F’s. These findings highlight the link between health-related behaviors and education outcomes, suggesting that education and public health professionals can find their respective education and health improvement goals to be mutually beneficial. Education and public health professionals might benefit from collaborating to achieve both improved education and health outcomes for youths.

The national YRBS is a biennial, school-based survey of U.S. high school students conducted by CDC. For the 2015 survey, a three-stage cluster sample design was used to produce a nationally representative sample of students in grades 9–12 who attended public and private schools (*6*). The school response rate was 69%, the student response rate was 86%, and the overall response rate (the school response rate multiplied by the student response rate) was 60%. Data were weighted based on sex, race/ethnicity, and school grade to adjust for nonresponse and oversampling of black and Hispanic students. The final data set included data from 15,624 students in grades 9–12.

School-level parental permission procedures were followed before survey administration, and participation was voluntary. Survey procedures were designed to protect students’ privacy by allowing for anonymous participation. Students completed the self-administered questionnaire during a single class period and recorded their responses on a computer-scannable booklet or answer sheet.

Academic achievement was measured with a question on self-reported letter grades in school: “During the past 12 months, how would you describe your grades in school?” Students could select one of the following response options: mostly A’s, mostly B’s, mostly C’s, mostly D’s, mostly F’s, none of these grades, and not sure. Data from additional questions were used to measure five dietary behaviors, three physical activity behaviors, two sedentary behaviors, seven substance use behaviors, five sexual risk behaviors, five violence-related behaviors, and three suicide-related behaviors. The dietary behaviors included (for the 7 days before the survey): ate breakfast on all 7 days; ate fruit or drank 100% fruit juices one or more times per day; ate vegetables one or more times per day; drank one or more glasses of milk per day; and did not drink a can, bottle, or glass of soda or pop. The physical activity behaviors included being physically active at least 60 minutes per day on 5 or more days during the 7 days before the survey, played on at least one sports team during the 12 months before the survey, and attended physical education classes on 1 or more days in an average week when they were in school. The sedentary behaviors included having watched television 3 or more hours per day on an average school day, and played video or computer games or used a computer for something that was not school work 3 or more hours per day on an average school day.

The substance use behaviors included current alcohol use (on at least 1 day during the 30 days before the survey); current marijuana use (one or more times during the 30 days before the survey); ever use of cocaine, ever use of heroin, ever use of methamphetamines, ever injection of any illegal drug, and ever took prescription drugs without a doctor’s prescription. The sexual risk behaviors included ever had sexual intercourse, had sexual intercourse with four or more persons, currently sexually active (had sexual intercourse during the 3 months before the survey), did not use a condom during last sexual intercourse, and did not use any method to prevent pregnancy during last sexual intercourse. The violence-related behaviors included having experienced, during the 12 months before the survey, physical violence by someone they were dating or going out with, sexual violence by someone they were dating or going out with, being bullied on school property, and being electronically bullied, and, during the 30 days before the survey, not going to school because of safety concerns. Finally, the suicide-related behaviors included having, during the 12 months before the survey, seriously considered attempting suicide, made a plan about how they would attempt suicide, and attempted suicide. Four additional questions on sex, race, ethnicity, and grade in school were used to create control variables for the statistical analyses.

Unadjusted prevalence estimates were calculated. Logistic regression models were used to determine whether the categorical variable of self-reported grades in school was associated with each health-related behavior while controlling for sex, race/ethnicity, and grade (9th, 10th, 11th, or 12th). Wald F p-values from the logistic regressions were used to determine statistically significant associations between overall self-reported letter grades in school and each behavior with an alpha level of 0.05. Comparisons of students with specific self-reported grades (mostly A’s, mostly B’s, or mostly C’s) against a combined referent group of students with mostly D’s/F’s were also assessed.

Unadjusted percentages showed a general gradient of association between self-reported letter grades and health behaviors (Table 1). After adjusting for sex, race/ethnicity, and grade level, overall self-reported grades in school were significantly associated with each behavior (p<0.05), except for physical education attendance (p = 0.6416) (Table 2). Students with mostly A’s, mostly B’s, or mostly C’s had significantly higher prevalence estimates for most protective health-related behaviors and significantly lower prevalence estimates for most health-related risk behaviors, including all substance use, sexual risk, violence-related, and suicide-related behaviors (Table 2). Prevalence estimates for students with mostly C’s were not significantly different from those for students with mostly D’s/F’s for two behaviors: ate vegetables one or more times per day during the past 7 days and watched television 3 or more hours per day on an average school day.

**TABLE 1:** Unadjusted weighted prevalence of health-related behaviors, by letter grades earned among high school students — National Youth Risk Behavior Survey, United States, 2015

Health-related behavior	% (95% CI)			
	Mostly A’s	Mostly B’s	Mostly C’s	Mostly D’s/F’s
**Dietary behavior**				
Ate breakfast on all 7 days*	45.4 (40.8–50.1)	35.2 (33.2–37.4)	28.7 (26.2–31.2)	18.8 (15.3–22.8)
Ate fruit or drank 100% fruit juices one or more times per day*	68.2 (65.0–71.2)	62.8 (60.8–64.7)	60.5 (57.1–63.7)	52.5 (47.9–57.2)
Ate vegetables one or more times per day^†^	68.0 (64.6–71.2)	60.0 (57.6–62.4)	55.1 (52.0–58.2)	54.2 (49.9–58.5)
Drank one or more glasses per day of milk*	42.8 (38.3–47.5)	35.7 (33.5–37.9)	33.8 (31.0–36.8)	28.2 (23.8–33.2)
Did not drink a can, bottle, or glass of soda or pop^§^	34.2 (30.1–38.6)	24.8 (22.3–27.4)	17.7 (15.3–20.4)	13.1 (10.2–16.7)
**Physical activity behavior**				
Physically active at least 60 minutes per day on 5 or more days^¶^	51.5 (47.2–55.8)	50.7 (47.8–53.6)	43.9 (40.9–47.0)	38.3 (33.8–43.1)
Played on at least one sports team**	66.9 (59.9–73.3)	58.9 (56.2–61.5)	48.6 (45.7–51.5)	36.7 (30.9–43.0)
Attended physical education classes on one or more days^††^	49.9 (43.1–56.7)	50.5 (43.8–57.1)	53.9 (48.8–58.8)	59.4 (52.2–66.1)
**Sedentary behavior**				
Watched television 3 or more hours per day^§§^	18.3 (15.2–21.9)	25.2 (23.3–27.3)	30.6 (28.2–33.2)	35.3 (30.2–40.7)
Played video or computer games or used a computer 3 or more hours per day^¶¶^	36.0 (32.2–40.0)	41.6 (39.2–44.1)	47.3 (44.0–50.5)	53.4 (49.1–57.7)
**Substance use**				
Currently drank alcohol***	24.3 (20.8–28.1)	34.6 (32.6–36.7)	40.4 (36.7–44.3)	51.6 (46.1–57.0)
Currently used marijuana^†††^	11.5 (9.4–14.0)	21.7 (19.2–24.5)	30.7 (27.8–33.7)	46.9 (41.4–52.5)
Ever used cocaine^§§§^	2.5 (1.8–3.6)	4.4 (3.5–5.5)	6.4 (5.2–7.8)	19.2 (14.8–24.6)
Ever used heroin^¶¶¶^	0.9 (0.5–1.6)	1.2 (0.8–1.8)	2.1 (1.2–3.6)	10.0 (6.8–14.4)
Ever used methamphetamines****	1.3 (0.8–2.0)	2.3 (1.7–3.1)	3.5 (2.6–4.7)	11.9 (8.9–15.7)
Ever injected any illegal drug^††††^	0.8 (0.5–1.3)	1.2 (0.7–2.1)	1.9 (1.2–3.0)	8.9 (6.3–12.6)
Ever took prescription drugs without a doctor’s prescription^§§§§^	10.7 (9.2–12.4)	16.7 (15.0–18.6)	21.7 (19.6–23.9)	34.1 (29.0–39.7)
**Sexual risk behavior**				
Ever had sexual intercourse	30.5 (26.1–35.3)	40.7 (37.2–44.2)	54.2 (50.9–57.4)	62.1 (55.7–68.1)
Had sexual intercourse with four or more persons^¶¶¶¶^	6.3 (4.4–8.9)	11.3 (9.8–13.1)	16.6 (14.6–18.9)	26.2 (19.8–33.7)
Currently sexually active*****	23.0 (19.8–26.6)	30.0 (27.3–32.9)	38.0 (35.4–40.6)	45.8 (40.2–51.5)
Did not use a condom during last sexual intercourse^†††††^	38.6 (33.5–44.0)	42.0 (38.5–45.6)	46.3 (41.2–51.6)	58.7 (48.7–68.0)
Did not use any method to prevent pregnancy during last sexual intercourse^†††††^	8.9 (6.8–11.5)	11.7 (9.1–15.0)	16.4 (12.8–20.9)	31.3 (23.3–40.7)
**Violence-related behavior**				
Experienced physical dating violence^§§§§§^	7.4 (5.9–9.3)	9.2 (8.0–10.5)	10.3 (8.9–12.0)	18.6 (14.5–23.7)
Experienced sexual dating violence^¶¶¶¶¶^	9.5 (7.8–11.6)	10.6 (9.3–12.0)	9.6 (8.0–11.5)	16.7 (12.1–22.5)
Were bullied on school property******	19.6 (17.5–21.9)	19.7 (18.0–21.6)	19.6 (17.2–22.3)	31.1 (27.5–34.9)
Were electronically bullied^††††††^	14.7 (13.3–16.1)	14.9 (13.2–16.8)	16.8 (14.8–18.9)	25.6 (21.8–30.0)
Did not go to school because of safety concerns^§§§§§§^	2.8 (1.9–4.1)	5.2 (4.3–6.2)	7.3 (5.9–9.1)	15.3 (12.1–19.2)
**Suicide-related behavior**				
Seriously considered attempting suicide******	14.1 (12.5–15.9)	15.7 (14.3–17.2)	21.7 (19.4–24.2)	36.0 (30.7–41.5)
Made a plan about how they would attempt suicide******	11.3 (9.4–13.5)	13.8 (12.5–15.3)	17.6 (15.3–20.1)	27.6 (23.4–32.2)
Attempted suicide^¶¶¶¶¶¶^	5.6 (4.4–7.1)	7.4 (6.5–8.4)	11.7 (9.9–13.9)	21.3 (17.4–25.8)

**Abbreviation:** CI = confidence interval.

* During the 7 days before the survey.

^†^ Includes green salad, potatoes (excluding French fries, fried potatoes, or potato chips), carrots, or other vegetables, during the 7 days before the survey.

^§^ Not including diet soda or diet pop, during the 7 days before the survey.

^¶^ Doing any kind of physical activity that increased their heart rate and made them breathe hard some of the time during the 7 days before the survey.

** Run by their school or community groups during the 12 months before the survey.

^††^ In an average week when they were in school.

^§§^ On an average school day.

^¶¶^ For something that was not school work on an average school day.

*** At least one drink of alcohol on at least 1 day during the 30 days before the survey.

^†††^ One or more times during the 30 days before the survey.

^§§§^ Any form of cocaine, such as powder, crack, or freebase, one or more times during their life.

^¶¶¶^ Also called “smack,” “junk,” or “China white,” one or more times during their life.

**** Also called “speed,” “crystal,” “crank,” or “ice,” one or more times during their life.

^††††^ Used a needle to inject any illegal drug into their body one or more times during their life.

^§§§§^ Such as OxyContin, Percocet, Vicodin, codeine, Adderall, Ritalin, or Xanax, one or more times during their life.

^¶¶¶¶^ During their life.

***** Had sexual intercourse with at least one person during the 3 months before the survey.

^†††††^ During last sexual intercourse among the 30.1% of students nationwide who were currently sexually active.

^§§§§§^ One or more times during the 12 months before the survey, being physically hurt on purpose (including being hit, slammed into something, or injured with an object or weapon on purpose) by someone they were dating or going out with among the 68.6% of students nationwide who dated or went out with someone during the 12 months before the survey. (Note: the prevalence of dating or going out with someone during the 12 months before the survey varies slightly for physical and sexual dating violence because of the differences in the number of usable responses to each violence question.)

^¶¶¶¶¶^ One or more times during the 12 months before the survey, being forced to do sexual things (including kissing, touching, or being physically forced to have sexual intercourse) they did not want to do by someone they were dating or going out with among the 69.1% of students nationwide who dated or went out with someone during the 12 months before the survey.

****** During the 12 months before the survey.

^††††††^ Including being bullied through e-mail, chat rooms, instant messaging, Websites, or texting during the 12 months before the survey.

^§§§§§§^ Did not go to school because they felt unsafe at school or on their way to or from school on at least 1 day during the 30 days before the survey.

^¶¶¶¶¶¶^ One or more times during the 12 months before the survey.

**TABLE 2:** Adjusted prevalence ratios* for health-related behaviors, by letter grades earned among high school students (using mostly D’s/F’s as the referent) — National Youth Risk Behavior Survey, United States, 2015

Health-related behavior	aPR (95% CI)			Wald F p-value
	Mostly A’s	Mostly B’s	Mostly C’s	
**Dietary behavior**				
Ate breakfast on all 7 days^†^	2.13 (1.89–2.41)	1.66 (1.46–1.89)	1.39 (1.23–1.56)	<0.0001
Ate fruit or drank 100% fruit juices one or more times per day^†^	1.28 (1.20–1.37)	1.17 (1.09–1.25)	1.12 (1.04–1.21)	<0.0001
Ate vegetables one or more times per day^§^	1.22 (1.13–1.32)	1.08 (1.01–1.16)	1.01 (0.94–1.09)	<0.0001
Drank one or more glasses per day of milk^†^	1.58 (1.35–1.84)	1.28 (1.12–1.47)	1.20 (1.04–1.39)	<0.0001
Did not drink a can, bottle, or glass of soda or pop^¶^	2.18 (1.79–2.65)	1.63 (1.35–1.96)	1.25 (1.01–1.54)	<0.0001
**Physical activity behavior**				
Physically active at least 60 minutes per day on 5 or more days**	1.37 (1.23–1.52)	1.33 (1.22–1.45)	1.14 (1.04–1.26)	<0.0001
Played on at least one sports team^††^	1.62 (1.45–1.82)	1.44 (1.32–1.57)	1.22 (1.11–1.34)	<0.0001
Attended physical education classes on one or more days^§§^	0.94 (0.85–1.04)	0.93 (0.82–1.06)	0.97 (0.88–1.07)	0.6416
**Sedentary behavior**				
Watched television 3 or more hours per day^¶¶^	0.54 (0.43–0.67)	0.72 (0.60–0.86)	0.84 (0.69–1.02)	<0.0001
Played video or computer games or used a computer 3 or more hours per day***	0.66 (0.57–0.76)	0.77 (0.68–0.87)	0.88 (0.77–0.99)	<0.0001
**Substance use**				
Currently drank alcohol^†††^	0.43 (0.37–0.50)	0.64 (0.57–0.71)	0.77 (0.67–0.89)	<0.0001
Currently used marijuana^§§§^	0.24 (0.19–0.29)	0.44 (0.38–0.52)	0.62 (0.54–0.72)	<0.0001
Ever used cocaine^¶¶¶^	0.14 (0.09–0.23)	0.22 (0.17–0.30)	0.33 (0.25–0.44)	<0.0001
Ever used heroin****	0.10 (0.05–0.21)	0.12 (0.08–0.20)	0.22 (0.11–0.43)	<0.0001
Ever used methamphetamines^††††^	0.12 (0.07–0.21)	0.20 (0.14–0.28)	0.31 (0.20–0.48)	<0.0001
Ever injected any illegal drug^§§§§^	0.11 (0.06–0.21)	0.15 (0.09–0.24)	0.24 (0.14–0.41)	<0.0001
Ever took prescription drugs without a doctor’s prescription^¶¶¶¶^	0.30 (0.23–0.38)	0.46 (0.38–0.56)	0.62 (0.52–0.73)	<0.0001
**Sexual risk behavior**				
Ever had sexual intercourse	0.47 (0.41–0.54)	0.61 (0.56–0.67)	0.82 (0.76–0.89)	<0.0001
Had sexual intercourse with four or more persons*****	0.24 (0.17–0.35)	0.40 (0.31–0.52)	0.56 (0.45–0.70)	<0.0001
Currently sexually active^†††††^	0.46 (0.40–0.53)	0.60 (0.53–0.67)	0.77 (0.70–0.86)	<0.0001
Did not use a condom during last sexual intercourse^§§§§§^	0.61 (0.50–0.74)	0.70 (0.58–0.84)	0.81 (0.68–0.97)	0.0001
Did not use any method to prevent pregnancy during last sexual intercourse^§§§§§^	0.29 (0.19–0.42)	0.38 (0.27–0.54)	0.54 (0.40–0.74)	<0.0001
**Violence-related behavior**				
Experienced physical dating violence^¶¶¶¶¶^	0.36 (0.25–0.51)	0.47 (0.36–0.62)	0.55 (0.41–0.75)	<0.0001
Experienced sexual dating violence******	0.49 (0.34–0.73)	0.62 (0.47–0.82)	0.59 (0.43–0.81)	0.0069
Were bullied on school property^††††††^	0.55 (0.45–0.67)	0.62 (0.52–0.72)	0.66 (0.54–0.80)	<0.0001
Were electronically bullied^§§§§§§^	0.45 (0.37–0.56)	0.56 (0.45–0.68)	0.69 (0.55–0.85)	<0.0001
Did not go to school because of safety concerns^¶¶¶¶¶¶^	0.20 (0.12–0.31)	0.35 (0.26–0.48)	0.51 (0.38–0.69)	<0.0001
**Suicide-related behavior**				
Seriously considered attempting suicide^†††††††^	0.35 (0.29–0.42)	0.43 (0.36–0.51)	0.64 (0.53–0.77)	<0.0001
Made a plan about how they would attempt suicide^††††††^	0.36 (0.28–0.46)	0.49 (0.41–0.58)	0.66 (0.53–0.83)	<0.0001
Attempted suicide*******	0.25 (0.19–0.33)	0.35 (0.27–0.47)	0.59 (0.45–0.78)	<0.0001

**Abbreviations:** aPR = adjusted prevalence ratio; CI = confidence interval.

* Logistic regression adjusted for sex, race/ethnicity, and grade (i.e., 9th, 10th, 11th, or 12th) in school.

^†^ During the 7 days before the survey.

^§^ Includes green salad, potatoes (excluding French fries, fried potatoes, or potato chips), carrots, or other vegetables, during the 7 days before the survey.

^¶^ Not including diet soda or diet pop, during the 7 days before the survey.

** Doing any kind of physical activity that increased their heart rate and made them breathe hard some of the time during the 7 days before the survey.

^††^ Run by their school or community groups during the 12 months before the survey.

^§§^ In an average week when they were in school.

^¶¶^ On an average school day.

*** For something that was not school work on an average school day.

^†††^ At least one drink of alcohol on at least 1 day during the 30 days before the survey.

^§§§^ One or more times during the 30 days before the survey.

^¶¶¶^ Any form of cocaine, such as powder, crack, or freebase, one or more times during their life.

**** Also called “smack,” “junk,” or “China white,” one or more times during their life.

^††††^ Also called “speed,” “crystal,” “crank,” or “ice,” one or more times during their life.

^§§§§^ Used a needle to inject any illegal drug into their body one or more times during their life.

^¶¶¶¶^ Such as OxyContin, Percocet, Vicodin, codeine, Adderall, Ritalin, or Xanax, one or more times during their life.

***** During their life.

^†††††^ Had sexual intercourse with at least one person during the 3 months before the survey.

^§§§§§^ During last sexual intercourse among the 30.1% of students nationwide who were currently sexually active.

^¶¶¶¶¶^ One or more times during the 12 months before the survey, being physically hurt on purpose (including being hit, slammed into something, or injured with an object or weapon on purpose) by someone they were dating or going out with among the 68.6% of students nationwide who dated or went out with someone during the 12 months before the survey. (Note: the prevalence of dating or going out with someone during the 12 months before the survey varies slightly for physical and sexual dating violence because of the differences in the number of usable responses to each violence question.)

****** One or more times during the 12 months before the survey, being forced to do sexual things (including kissing, touching, or being physically forced to have sexual intercourse) when they did not want to do by someone they were dating or going out with among the 69.1% of students nationwide who dated or went out with someone during the 12 months before the survey.

^††††††^ During the 12 months before the survey.

^§§§§§§^ Including being bullied through email, chat rooms, instant messaging, Websites, or texting during the 12 months before the survey.

^¶¶¶¶¶¶^ Did not go to school because they felt unsafe at school or on their way to or from school on at least 1 day during the 30 days before the survey.

******* One or more times during the 12 months before the survey.

## Discussion

Among U.S. high school students, healthy eating and physical activity were associated with higher self-reported letter grades, whereas sedentary, substance-use, sexual risk, violence-related, and suicide-related behaviors were associated with lower self-reported grades. This relationship, which appears similar for both lifetime and more recent behaviors (i.e., behaviors that occurred one or more times during a student’s life and behaviors that occurred during the previous 7 days, 30 days, 3 months, or 12 months), is consistent with findings of other reports (*1*–*4*,*7*). A 2013 systematic review examining 25 years of evidence related to academic achievement and health-related behaviors across a wide range of ages and grade levels found that 96.8% of reviewed cross-sectional studies and 93.1% of longitudinal studies identified statistically significant associations between some form of academic achievement and behaviors related to physical activity, nutrition, substance use, sexual risk, or violence (*1*). With no assessment of self-reported academic performance on YRBS since 2009, this report of 2015 data from a nationwide sample of high school students supports earlier findings and offers updated, nationally representative findings as evidence of a continuing association between health-related behaviors and academic achievement.

Although causation cannot be inferred from the current analysis, causal relationships are believed to exist in both directions between education and health (*1*,*8*). Longitudinal studies in the 2013 literature review concluded that less engagement in health risk behaviors among persons aged 10–18 years leads to higher achievement later in life, and that earlier academic achievement during the same period leads to less health risk behaviors later in life (*1*). Education is commonly viewed as an important social determinant of health, leading some health professionals to measure and target education-related outcomes in health-focused programming (*2*,*7*). Conversely, some educational researchers have advocated addressing health risk behaviors and related disparities as a key approach to closing academic achievement gaps among youths (*9*).

Highlighting the association between education and health might facilitate the establishment of partnerships between health agencies and education agencies, many of which are well positioned to support health programs, in part because of existing infrastructure to support educational interventions, health services, and family and community involvement. U.S. high schools enroll approximately 16.5 million youths,* and schools provide the physical and social environment in which youths spend much of their day at a key phase of life when many youths engage in risk behaviors. Schools face tremendous pressure to reach educational goals. These findings, combined with existing evidence that improved academic achievement outcomes can be seen from health programs based on the coordinated school health or Whole School, Whole Community, Whole Child models, suggest that efforts to improve health among students might contribute to achievement of those educational goals (*7*).

The findings in this report are subject to at least four limitations. First, because data analyzed in this report are cross-sectional, findings show only associations and cannot demonstrate causality in either direction. Second, this study does not address potential confounding (e.g., the extent to which both health behaviors and educational outcomes might result from other factors such as family context and neighborhood setting). However, several studies included in the 2013 review found that the association between health-related behaviors and education outcomes can be seen even when accounting for family and community contextual variables (*1*). Third, these data represent only youths who attend school and are not representative of all youths in this age group. Data from 2012 indicated that approximately 2.9% of youths aged 16 and 17 years in the United States had dropped out of high school^†^; such youths are not represented in this report. Finally, CDC cannot determine the extent to which respondents might overreport or underreport behaviors or grades in school; however, YRBS questions have demonstrated good test-retest reliability (*6*).

School health interventions can promote positive health behaviors by 1) offering students opportunities to practice healthy behaviors; 2) increasing student knowledge and skills through school nutrition programs and services, physical education, and comprehensive health education (including sexual health education); 3) enhancing protective factors such as school connectedness or parent engagement; and 4) shaping school health services and environments more broadly (*9*,*10*). School health programs based on the Whole School, Whole Community, Whole Child or coordinated school health models (https://www.cdc.gov/healthyyouth/wscc/) that include safe, supportive environments and engagement from communities and families as key components, have been linked to improved academic achievement outcomes among students (*2*,*7*). Such evidence suggests that education and public health professionals have a shared interest in promoting student health and that collaborative efforts have the potential to make important strides in improving the health and academic achievement of youths.

**Summary**

**What is already known about this topic?**

Studies have shown links between health-related behaviors and educational outcomes such as grades, test scores, and other measures of academic achievement; however, many of these studies have used samples that are not nationally representative or are out of date.

**What is added by this report?**

Analyses of nationwide 2015 Youth Risk Behavior Survey data (controlling for sex, race/ethnicity, and grade in school) reveal that high school students who received mostly A’s, mostly B’s, or mostly C’s had significantly higher prevalence estimates for most protective health-related behaviors and significantly lower prevalence estimates for most health-related risk behaviors compared with students with mostly D’s/F’s.

**What are the implications for public health practice?**

School health interventions can promote positive health behaviors and improve both health and academic outcomes for students. Evidence suggests that educational and public health institutions have a shared interest in promoting student health and that collaborative efforts have the potential to make important strides in improving the health and academic achievement of youths.

## Footnotes

* https://www.census.gov/data/tables/2015/demo/school-enrollment/2015-cps.html.

† https://nces.ed.gov/pubs2015/2015015.pdf.
